# Crosstalk between lipid metabolism and macrophages in atherosclerosis: therapeutic potential of natural products

**DOI:** 10.3389/fcvm.2025.1529924

**Published:** 2025-03-03

**Authors:** Taoming Qian, Donghao Guo, Lu Sun, Ming Chi, Xiaoshuang Ma, Juan Jin

**Affiliations:** ^1^ Graduate School, Heilongjiang University of Chinese Medicine, Harbin, China; ^2^ Department of Cardiovascular Disease 1, The First Affiliated Hospital of Heilongjiang University of Chinese Medicine, Harbin, China

**Keywords:** atherosclerosis, lipids, macrophages, natural drug, metabolism

## Abstract

Atherosclerosis is a highly prevalent cardiovascular condition that affects individuals worldwide. Despite ongoing research into its treatment and prevention, atherosclerotic cardiovascular disease continues to exhibit high morbidity and mortality rates. The accumulation of low-density lipoprotein cholesterol is considered a major contributor to the development of atherosclerosis, with abnormalities in lipid metabolism playing a significant role in its pathogenesis. Lipid metabolism and macrophage function are intricately interconnected, with lipid metabolism being influenced by macrophage inflammatory responses, while macrophage activity is regulated by alterations in lipid metabolism. The interaction between these two processes plays a critical role in the progression of atherosclerosis. Natural products have shown considerable promise in treating a variety of diseases, including atherosclerosis. Moreover, the modulation of lipid metabolism and macrophage crosstalk represents a key mechanism through which natural products may exert their effects. This research aims to provide new insights into the current state of research on the role of natural products in regulating this pathway and the interplay between lipid metabolism and macrophages in the context of atherosclerosis, offering potential directions for the future.

## Graphical Abstract

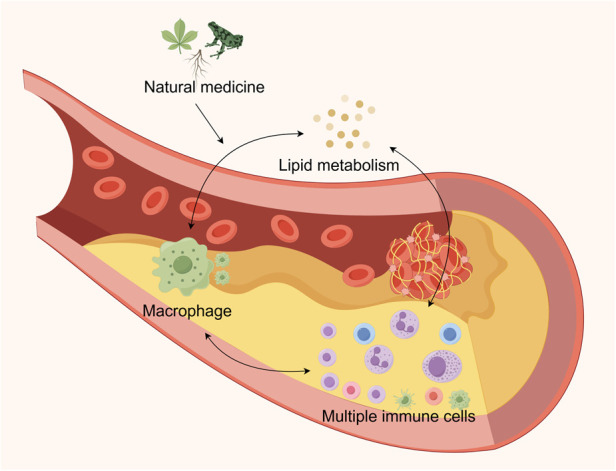

## 1 Introduction

Atherosclerosis is a condition characterized by the narrowing of arteries due to endothelial dysfunction, lipid deposition in the vascular intima, and the subsequent recruitment of inflammatory cells, including monocytes and macrophages (1). Globally, atherosclerosis plays a significant role in the development of cardiovascular diseases, such as stroke and myocardial infarction (2). Cardiovascular disease (CVD) is currently the leading cause of death worldwide, with its incidence rising continuously over the past few decades (3). Atherosclerotic cardiovascular disease is estimated to account for nearly two-thirds of fatalities related to atherosclerosis globally (4). Ischemic heart disease and stroke are among the leading causes of death in this category. In Europe, CVD claims approximately 4 million lives annually, with ischemic heart disease contributing 44% and stroke 25% of these deaths (5). In China, where ischemic heart disease and stroke are the primary causes of death, diseases linked to atherosclerotic cardiovascular disease follow a similar pattern (6).

Atherosclerosis develops as a result of abnormalities in lipid metabolism, with low-density lipoprotein (LDL) accumulation playing a significant role in its pathogenesis. It is widely believed that LDL is a primary causative agent of atherosclerosis (7). However, abnormalities in other lipids may also compromise arterial health. A study revealed that, even in individuals undergoing treatment to lower LDL-C, hypertriglyceridemia remains a serious risk factor for atherosclerotic cardiovascular disease (ASCVD). Numerous large-scale observational studies have consistently shown an association between hypertriglyceridemia and ASCVD. The presence of low high-density lipoprotein (HDL) cholesterol further complicates this relationship (8).

Macrophages play a crucial role in the development of atherosclerosis. As the predominant immune cell population within arterial plaques, macrophages are believed to be essential for both the immune response and the progression of atherosclerosis (2, 4). The primary sources of macrophages are resident tissues and circulating monocytes. These macrophages are attracted to lesions by binding to activated endothelial cells (EC) and migrating into the subendothelial space (5). In advanced plaques, macrophage proliferation becomes the primary replenishment mechanism (6). A key feature of early atherosclerotic lesions is the ability of macrophages within plaques to engulf lipid particles and differentiate into foam cells (7). These foam cells initiate a cascade of inflammatory responses, leading to increased retention of lipoproteins, alteration of the extracellular matrix (ECM), and sustained chronic inflammation (8). Moreover, foam cell necrosis is further exacerbated by modified LDL particles, such as oxidized LDL (ox-LDL), which may result in the formation of necrotic cores. This is a common feature of advanced plaque instability, which can lead to acute, life-threatening cardiovascular events and plaque rupture.

Natural products have become essential in the treatment of a variety of diseases, including neurological disorders, mental health issues, and cancers (9–13). Due to their low side effect profile and multi-target therapeutic potential, they have attracted considerable attention in recent years. Natural products are derived from various sources, with plants being the primary source of natural medicines. Atherosclerosis research has primarily focused on plant-based remedies (14–16). However, it is important to also acknowledge the significance of natural medicines derived from animal and microbial sources, and further investigation into their potential therapeutic benefits is warranted. This paper provides a systematic summary of studies conducted over the past five years, outlining the role of various lipid metabolism abnormalities in atherosclerosis and the interaction between lipid metabolism and macrophages. To generate new insights for future research, we conclude by reviewing studies on several natural compounds that have been shown to improve atherosclerosis by modulating the interaction between these two factors.

## 2 Different types of abnormal lipid metabolism affect atherosclerosis

### 2.1 HDL

High-density lipoprotein (HDL) is a critical component of the lipoprotein class in blood and plays a vital role in the intricate network of lipid metabolism. Often referred to as “good cholesterol,” HDL's significant impact on cardiovascular health underscores its critical role (17). These unique lipoprotein particles, which are smaller and denser than other types, follow the general structural framework of lipoproteins, consisting of a hydrophobic core encased by a hydrophilic outer shell. Numerous studies have emphasized the benefits of HDL in maintaining cardiovascular health (18). It achieves this by actively supporting endothelial cell function and efficiently preventing the oxidation of LDL, thereby offering protection against the formation of atherosclerotic plaque. Additionally, HDL stimulates the synthesis of nitric oxide, a potent vasodilator that enhances the elasticity and flexibility of blood vessels, while also contributing to the regulation of blood pressure (19). Scientific research consistently demonstrates a strong correlation between higher HDL cholesterol levels and a reduced risk of developing cardiovascular diseases. As a result, maintaining or increasing HDL levels has become one of the most crucial strategies for the prevention of cardiovascular diseases (20).

### 2.2 LDL

The primary function of LDL, a critical lipoprotein found in the blood, is to transport triglycerides (TG) and cholesterol from the liver to peripheral tissues. Due to its strong association with an increased risk of cardiovascular disease, LDL is often referred to as “bad cholesterol.” Elevated LDL levels in the blood have been shown to correlate with a heightened risk of cardiovascular disease (21). In terms of morphology, LDL particles are less dense and more prominent than other lipoproteins, such as HDL. Their core is composed of densely packed hydrophobic components, including triglycerides and cholesterol esters, while the periphery is surrounded by a monolayer membrane consisting of phospholipids, apolipoproteins, and free cholesterol. Among these, ApoB-100 is the major apolipoprotein component of LDL and plays a critical structural and functional role (22). The primary function of LDL is to ensure the accurate delivery of cholesterol to cells that require it for various physiological processes, such as hormone synthesis, bile acid production, and cell membrane formation. However, complications arise when LDL levels fall outside the normal range. Atherosclerosis, a condition characterized by the accumulation of plaques in the arteries, is triggered by the deposition of excess cholesterol in the artery walls due to elevated LDL levels. These plaques not only progressively enlarge and narrow the artery lumen but also harden the arterial walls, thereby reducing blood flow and significantly increasing the risk of major cardiovascular events, including stroke and myocardial infarction (23). Furthermore, LDL cholesterol has the potential to undergo oxidation within the artery walls. This modified form of LDL is even more harmful to the vasculature. When ox-LDL particles are taken up by macrophages in the artery wall, foam cells are formed, marking a critical step in the development of atherosclerotic plaques. This complex cascade of biochemical events accelerates the progression of atherosclerosis and poses a significant threat to cardiovascular health (20, 24).

### 2.3 Medium-density lipoprotein and very low-density lipoprotein

The body naturally produces Medium-density lipoprotein (MDL), a class of lipoproteins, during the breakdown of very low-density lipoprotein (VLDL). Its particle size and density fall between those of VLDL and LDL, forming a distinct intermediate lipoprotein (25). In the dynamic lipid circulation, MDL plays a temporary role in lipid transport. Triglyceride-rich VLDL particles interact with lipoprotein lipase to generate MDL. This lipoprotein consists of a complex mixture of phospholipids, cholesteryl esters, triglycerides, and various apolipoproteins, which together contribute to its unique physiological and metabolic properties. MDL, however, follows two distinct pathways rather than a single fate within the body. Through receptor-mediated endocytosis, certain MDL particles are taken up by the liver, where they undergo further metabolic processing (26). In the liver, MDL can either be directly converted back into VLDL or be transformed into LDL by lipoprotein lipase. This conversion pathway positions MDL as a precursor of LDL. Cholesteryl ester transfer protein (CETP), a hepatic glycoprotein, facilitates the exchange of TG in VLDL for cholesteryl esters in LDL and HDL particles. This exchange enriches VLDL and LDL particles with cholesteryl esters, while HDL particles become enriched in TG, and VLDL and LDL particles are depleted of TG, while HDL particles lose cholesteryl esters (27). Consequently, MDL levels in the blood have become an important indicator of health. Elevated MDL levels are often associated with various metabolic disorders, including dyslipidemia. Proper lipid management is essential to maintaining cardiovascular health (24). This can be achieved through lifestyle modifications aimed at regulating MDL levels, preventing its conversion into “bad cholesterol,” and reducing the risk of atherosclerosis. These interventions may include dietary adjustments, increased physical activity, and, when necessary, pharmaceutical treatment (24).

## 3 Interactions between lipid metabolism and macrophages

**Figure 1:**
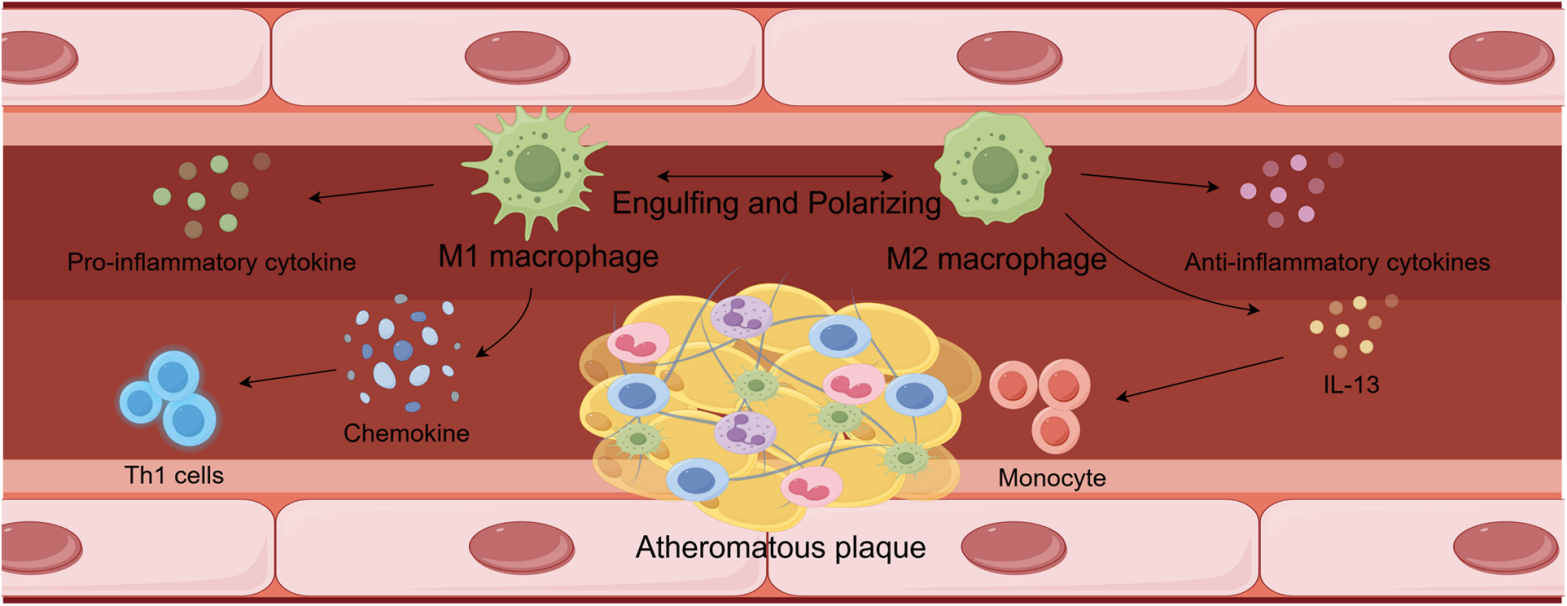
Interactions between lipid metabolism and macrophages.

Abnormalities in lipid metabolism are key factors in the development and progression of atherosclerosis, occurring throughout the course of the disease. Macrophages interact with lipid metabolism and are intricately involved in lipogenesis, catabolism, uptake, and transport (28). Additionally, the regulation of cholesterol metabolism and fatty acid β-oxidation are interconnected pathways that facilitate crosstalk between the two, as shown in Figure 1.

High concentrations of pro-inflammatory cytokines, including IL-6, IL-12, IL-23, TNF-α, and IL-1β, can be produced by M1 macrophages, maintaining an inflammatory response in the vascular environment. They can also release modest quantities of IL-10 and Th1 recruitment-associated chemokines including CXCL-9, CXCL-10, and CXCL-11 at the same time. The persistence of these proinflammatory responses can worsen the severity of atherosclerotic lesions. In atherosclerotic lesions, high concentrations of M2 macrophages were able to lower the inflammatory response by releasing anti-inflammatory molecules. Furthermore, M2 phenotypes produced by IL-13 were able to reduce VCAM-1-mediated monocyte recruitment and decrease collagen concentrations in lesions. These modifications encourage atherosclerosis to get better. Ultimately, M2-type macrophages were able to resolve inflammation and prevent necrotic cores from forming in atherosclerotic lesions by phagocytosing M1-type macrophages.

### 3.1 Lipogenesis

The initiation of lipid metabolism, known as lipogenesis, is closely associated with the regulation of macrophages. Enzymes such as fatty acid synthase (FAS) and acetyl-CoA carboxylase (ACC) catalyze the conversion of acetyl-CoA into fatty acids, which are then further modified by desaturases and/or elongases. These fatty acids can be esterified to form cholesteryl esters or 3-phosphoglycerol, which are subsequently converted into triglycerides and cholesterol, respectively. M2-type macrophages are linked to substances that influence lipid metabolism, while M1-type macrophages are primarily associated with lipid metabolism through the sterol regulatory element-binding protein (SREBP) pathway (28).

Through ACC and FAS, the transcription factor SREBP regulates the early stages of adipogenesis (28). The SREBP signaling pathway in adipogenesis plays a critical role in the lipid composition of macrophages and in their phagocytic activity, with the isoform SREBP-1a being highly expressed in macrophages (29, 30). The phagocytosis of pathogen-stimulated macrophages requires interactions between membrane lipid rafts and the actin cytoskeleton, and SREBP-1a serves as a key downstream effector in the TLR4-mTORC1 signaling axis (29). Furthermore, cytokines produced by M1-type macrophages are indirectly linked to SREBP and adipogenesis. Notably, the levels of pro-inflammatory cytokines such as TNF-α and IL-1β were significantly reduced in macrophages following FAS knockdown (31). Additionally, SREBP-1a is essential for the partial production of IL-1β induced by lipopolysaccharide (LPS) (30). SREBP can also trigger the synthesis of caspase-11, an inflammatory mediator that interacts with SREBP processing through site-1 protease (S1P) to activate SREBP-1a. In response to LPS, the caspase-11/S1P pathway promotes the activation of SREBP-1, which, in turn, regulates the subsequent activation of macrophages (32). Environmental pollutants have been shown to drive macrophages toward an inflammatory phenotype. Specifically, macrophages with unaltered LDL exhibit increased lipid accumulation, and exposure to aqueous PM2.5 promotes classical macrophage activation (33).

In contrast, certain compounds can influence lipid metabolism in M2-type macrophages, promoting their polarization toward the M2 phenotype. For instance, in the epididymal white adipose tissue (EWAT) of ob/ob mice, ursodeoxycholic acid (UDCA), a bile acid, was shown to reduce lipogenesis while enhancing the number of M2 macrophages (34). Similarly, the pro-inflammatory activity of macrophages was reduced by SO1989, a novel oleanolic acid derivative. This compound significantly alleviated chronic inflammation in high-fat diet (HFD) mice and restored M2 polarization in peritoneal macrophages (PM) and adipose tissue macrophages (ATM) that had been impaired by HFD (35).

### 3.2 Lipolysis

Three enzymes—adipose triglyceride lipase (ATGL), monoacylglycerol lipase (MAGL), and lysosomal acid lipase (LAL)—are closely associated with macrophages and lipolysis. In macrophage lipid droplets, ATGL plays a crucial role in the breakdown of triglycerides. Elevated ATGL-mediated lipolysis leads to impaired lipid retention, which causes a decrease in lipid droplets (36). At the same time, ATGL can prevent the generation of cytokines, such as IL-6, induced by LPS (37). While LPS enhances lipid storage, it suppresses lipolysis by reducing ATGL expression in macrophages (38). In response to reduced lipolysis, ATGL-deficient macrophages increase fatty acid uptake, but phagocytosis occurs only when sufficient fatty acids are available to generate ATP (39). The enzyme MAGL catalyzes the production of glycerol and free fatty acids from monoacylglycerol, and it suppresses autophagy while promoting inflammatory responses in macrophages (40). Finally, LAL regulates both mitochondrial oxidative respiration and macrophage polarization. Inhibition of LAL activity promotes M2-type macrophage polarization and prevents M1 polarization (41). Thus, lipolysis and macrophage polarization are interrelated.

### 3.3 Lipid uptake and transport

The CD36 receptor and PPAR-γ are primarily involved in lipid absorption and transport in macrophages. Macrophages are among the cell types that express the CD36 receptor, also known as glycoprotein IV (42). This receptor plays a role in the phagocytosis of apoptotic cells and the uptake of oxidized lipids and ox-LDLs (43, 44). Increased binding of ox-LDL to the CD36 receptor in macrophages generally promotes the development and progression of atherosclerotic plaques. The experimental autoimmune encephalomyelitis (EAE) model highlights the importance of CD36 in myelin debris clearance, where it reduces inflammation through macrophage- and microglia-mediated myelin removal. From a genetic standpoint, chromosome 7q11.2 contains a polymorphic gene that encodes CD36 (45). However, suppression of CD36 can sometimes lead to increased inflammation. Oxidized high-density lipoprotein (ox-HDL) induces CD36 palmitoylation and lipid uptake in macrophages, similar to that of ox-LDL (46). Consequently, the accumulation of cholesterol and oxidized lipids promotes the transformation of monocytes into foam cells (47). PPAR-γ influences macrophages through CD36, which upregulates CD36 receptor expression, enhances ox-LDL uptake, and promotes macrophage differentiation (48). Moreover, a deficiency in fatty acid-binding protein (FABP-5, for example) promotes fat storage through the PPAR-γ pathway, primarily affecting adipogenesis and fatty acid β-oxidation (FAO). *in vivo* suppression of FABP-5 exacerbates allergic asthma in a model of ovalbumin-induced allergic airway inflammation. Notably, excess oleic acid worsens allergic asthma by promoting FABP-5-dependent M2 polarization (49). FABP-5 has also been implicated in hepatic inflammation (50). These findings suggest that disrupted fatty acid homeostasis can impair macrophage metabolism and consequently affect surrounding tissues.

### 3.4 Cholesterol metabolism

M2-like macrophages and foam cells are involved in the uptake and transport of circulating cholesterol from the vasculature to the excretory system. Cholesterol is a crucial precursor for the synthesis of cell signaling molecules. Several factors that regulate macrophage inflammatory responses and cholesterol levels include SREBP, PPAR, and liver X receptor (LXR). The SREBP-2 isoform is associated with cholesterol metabolism in macrophages, and SREBPs facilitate cholesterol production (50, 51). In TNF-activated macrophages, SREBP-2 promotes targeting and interferon responsiveness of the mevalonate pathway (51). miR-33, a microRNA involved in SREBP signaling, also contributes to the expression of pro-inflammatory and anti-inflammatory genes in M1 and M2 macrophages, respectively (52). Moreover, in macrophages, both SREBP and miR-33 inhibit cholesterol efflux through the ATP-binding cassette transporter protein A1 (ABCA1) (50, 52). The effects of SREBP on inflammatory genes and cholesterol metabolism may play a role in the development of various related disorders, including atherosclerosis, a condition where foam cells are essential. In fact, alterations in cholesterol metabolism caused by the loss of SCAP, which regulates SREBP activity, prevent cholesterol export and induce pro-inflammatory M1 polarization in adipose tissue macrophages (53). Through transporter proteins (such as ABCA1) and apolipoproteins, the LXR promotes the efflux of cholesterol (54). A recent study demonstrated that caveolin-1, a multifunctional membrane protein required for phagocytosis, enables LXR to regulate cytokine synthesis and cholesterol metabolism in response to LPS (54, 55). Research also indicates that LXR activation polarizes M1 macrophages and increases IFN-γ, which could explain the decline in malignancy (51). However, the type of macrophage, the tissue involved, and the LXR subtype can all influence the activation or inhibition of LXR (52, 53).

Another transcription factor that influences cholesterol metabolism is PPAR, although it is found at lower concentrations in macrophages compared to other tissues like adipose tissue. PPAR-α and PPAR-γ subtypes induce cholesterol efflux by upregulating ABCA1 expression in macrophages through LXR-α (54). Since PPARγ suppresses LPS- and IFN-induced genes in macrophages, activating PPARγ with its agonist, rosiglitazone, may exert anti-inflammatory effects (55). Treatment with rosiglitazone consistently prevented cholesterol and cholesteryl ester accumulation in LPS-treated macrophages (56). In macrophages, LPS decreases the expression of PPAR-γ, PPAR-*δ*, and LXR-α. The reduced expression of ABCA1 contributes to the effects of LPS on cholesterol homeostasis, including the suppression of cholesterol efflux (55, 57). These findings suggest that in macrophages, LPS and PPAR have opposing actions. Adipocyte enhancer-binding protein 1 (AEBP1) plays a role in cholesterol metabolism through PPAR-γ and the inflammatory response via NF-kB in LPS-treated macrophages (57, 58). However, the PPAR-γ/AEBP1 pathway is not solely responsible for the inflammatory effects of LPS and its impact on cholesterol metabolism. The retinoid X receptor (RXR) may be an additional factor influencing how PPAR affects cholesterol metabolism. Previous studies have shown that activation of PPAR-γ, PPAR-α, and RXR prevents cholesterol and cholesteryl ester accumulation in macrophages in response to LPS (56). However, further research is needed to fully understand the cholesterol regulation network (59, 60).

Fatty acids undergo catabolism in the fatty acid oxidation process, which produces acetyl-CoA that is used in the mitochondrial tricarboxylic acid (TCA) cycle. An important enzyme, carnitine palmitoyltransferase 1 (CPT1), transports cytoplasmic fatty acids into mitochondria for further FAO processing. The inhibition of CPT1 in macrophages is linked to the progression of atherosclerosis, as it increases the production of CD36, a protein involved in LDL absorption (60). Moreover, when PPARα, an upstream regulator of CPT1, activates FAO, the levels of cholesteryl esters are reduced in TNFα-treated macrophages (61). FAO represents a viable therapeutic target since it is regulated in relation to overall fat/cholesterol levels and lipid uptake in macrophages (60).

For macrophage functions such as M2 polarization, inflammatory responses, and phagocytosis, FAO provides energy from lipids. IL-4 induces higher expression of CPT1, acyl-CoA dehydrogenase, and enoyl-CoA hydratase, resulting in increased FAO and fatty acid uptake (62). M1 macrophages primarily use glucose as their energy source, whereas M2 macrophages likely utilize fatty acids. M1 macrophages stimulated by IFN-γ/LPS exhibit enhanced glucose uptake, but not FAO. In contrast, M1 macrophages activated by LPS show enhanced glucose uptake but not FAO (62). Nevertheless, FAO also plays a role in the inflammatory response of M1 macrophages by contributing to the generation of pro-inflammatory cytokines (IL-1β, TNF-α, IL-6, and IL-12). Etomoxir, an inhibitor of CPT1, reduces cytokine production in a CPT1-dependent manner (63–65). Recent studies, however, have shown that high doses of etomoxir, which inhibit CPT1, significantly impact overall CoA homeostasis, suggesting that the polarization of IL-4-activated M2 macrophages may involve mechanisms beyond CPT1 (66). Factors such as PPARγ coactivator 1 (PGC1) and signal transducer and activator of transcription (STAT) also regulate FAO and CPT1 (62, 65, 67). Additionally, genetic studies have demonstrated the role of CPT1 in macrophage function. A recent study showed that phagocytic activity was enhanced in RAW264.7 macrophages expressing CPT1. Phagocytosis was reduced when CPT1 was knocked down using an adenoviral shRNA vector, while macrophages overexpressing CPT1 exhibited increased phagocytic activity (68). Overall, FAO plays a significant role in macrophage function, although experimental outcomes may vary.

## 4 Macrophages influence atherosclerosis progression

### 4.1 Macrophage source

Atherosclerotic plaques contain four primary types of macrophages: monocytes, locally proliferating macrophages, intimal vascular smooth muscle cells (VSMCs), and bone marrow-derived cells (69). Due to the diverse origins of these macrophages, their roles in atherosclerosis are complex. First, plaque-prone areas serve as a key site for macrophage recruitment. Moreover, extra-marrow hematopoiesis continuously generates monocytes, which subsequently differentiate into macrophages (70). Second, localized macrophage accumulation in plaques persists, particularly in advanced atherosclerotic lesions, where up to 87% of the total lesion area is occupied by proliferating macrophages (71–73). Furthermore, endothelial VSMCs have the potential to transdifferentiate into macrophage-like cells and exhibit considerable plasticity. Through phenotypic switching, VSMCs can give rise to macrophage-like cells, mesenchymal stromal/stem cells, and chondropoietic cells. On the one hand, VSMCs can internalize lipoproteins and transform into foam cells, thereby contributing to the progression of atherosclerosis due to their remarkable plasticity (74). Finally, bone marrow-derived monocytes and aortic macrophages originating from yolk sacs play important roles in maintaining homeostasis (75–77).

### 4.2 Effects of different macrophage subtypes

**Figure 2:**
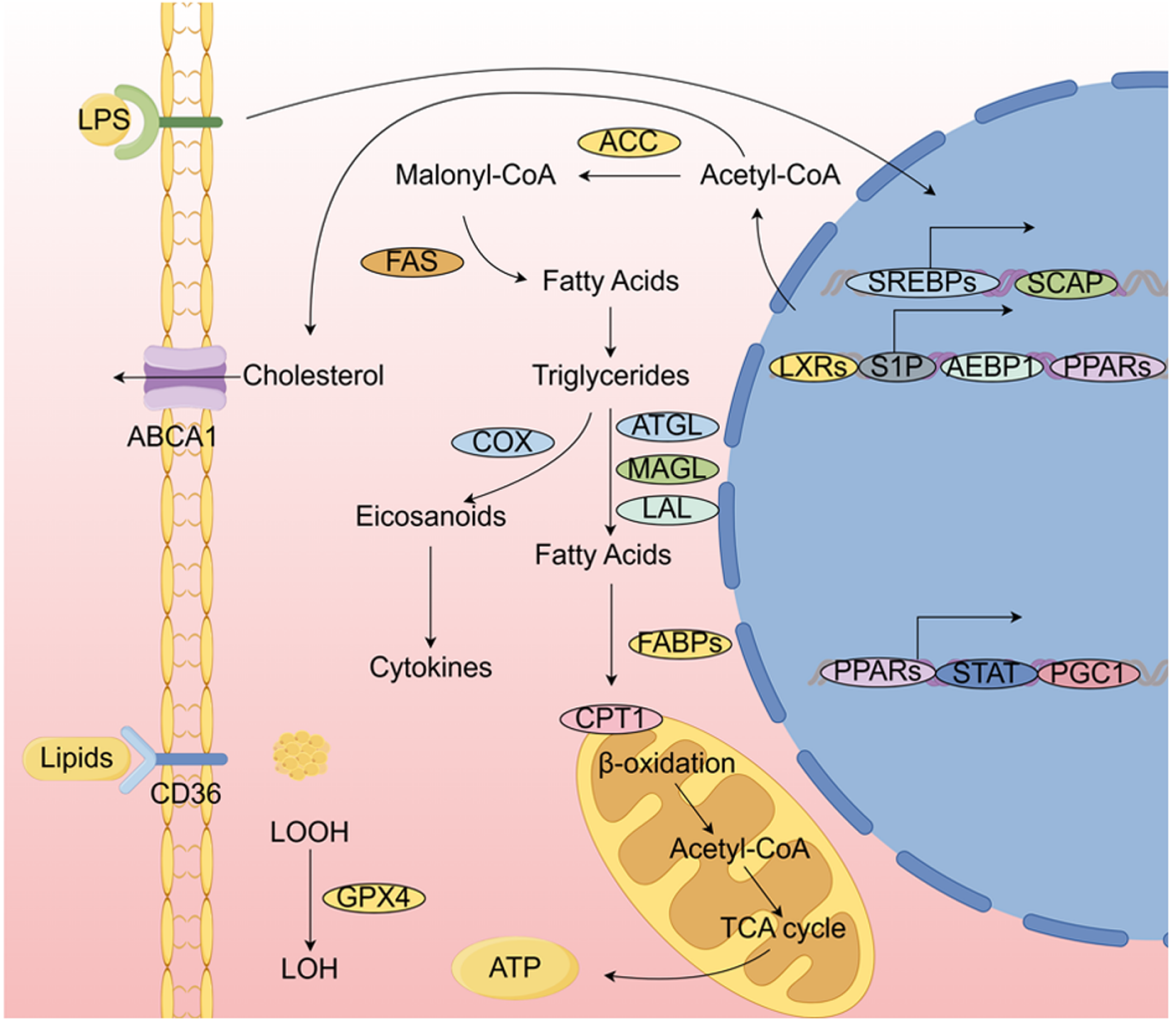
Pathways by which M1-type and M2-type macrophages influence atherosclerosis.

Although M1 and M2 are the most prevalent subtypes of macrophages, other subtypes, including Mox, Mhem, and M4, also exist. As atherosclerosis research progresses, the classification of macrophages is being refined. Recent studies utilizing RNA-seq technology have provided new insights into the categorization of macrophages in atherosclerosis. These studies identified five distinct groups: foamy Trem2 macrophages, resident-like macrophages, inflammatory macrophages, interferon-induced macrophages, and the recently discovered cavity macrophages (78, 79). This classification is critical for further investigating the epigenetic inheritance of macrophages in the context of atherosclerosis. While much of the current research continues to focus on the classical classification of macrophage subtypes, with M1 and M2 macrophages remaining central research topics, as depicted in Figure 2.

Lipogenesis involves acetyl coenzyme A, FAS, and acetyl coenzyme A carboxylase (ACC). When traditionally activated macrophages are exposed to lipopolysaccharide (LPS) and Toll-like receptor 4 (TLR4) pathways, lipogenesis is upregulated. Cholesterol, which can be further esterified and stored in lipid droplets, is synthesized from acetyl coenzyme A. The metabolism of fatty acids and cholesterol, along with their effects on macrophage polarization, inflammation, phagocytosis, and cholesterol efflux, is regulated by various transcription factors and cofactors. Before being incorporated into lipid droplets and organelles, lipids are absorbed by CD36. The catabolic process that converts triglycerides into fatty acids involves the lipases ATGL, MAGL, and LAL. For instance, this process releases lipid mediators, such as arachidonate-like acids (e.g., prostanoids), through the action of cyclooxygenase (COX). To produce ATP, free fatty acids are transported into mitochondria by fatty acid-binding protein (FABP). Acetyl coenzyme A is released by (CPT1 to initiate the TCA. The regulation of genes involved in lipid metabolism and their impact on macrophage function involves a range of transcription factors and their associated cofactors.

M1 macrophages are generally protective, producing reactive oxygen species to scavenge pathogens, such as bacteria, fungi, and viruses, thereby safeguarding the organism from attack (80). This is achieved by activating the nicotinamide adenine dinucleotide phosphate (NADPH) oxidase complex, which induces tissue damage (80). However, M1 macrophage-induced inflammation in atherosclerosis leads to tissue damage and impairs wound healing, both of which accelerate the development of atherosclerotic plaques (81, 82). M1 macrophages produce high levels of pro-inflammatory cytokines, including IL-6, IL-12, IL-23, TNF-α, and IL-1β, which sustain an inflammatory response in the vascular environment (83). In addition, low levels of IL-10 are produced, along with chemokines linked to Th1 recruitment, such as CXCL-9, CXCL-10, and CXCL-11 (84). The progression of atherosclerotic lesions may worsen as long as these pro-inflammatory responses persist (85).

In contrast, M2 macrophages suppress pro-inflammatory responses. Their functions include modulating inflammation, clearing apoptotic cells, promoting fibrosis and angiogenesis, and facilitating tissue regeneration (86). M2 macrophages are further categorized into three subtypes: M2a, M2b, and M2c, each activated by different cytokines. M2a macrophages are activated by increased levels of IL-4 and IL-13. Upon activation, they produce pro-fibrotic factors such as TGF-*β* and fibronectin, contributing to tissue regeneration (87). M2b macrophages are activated by ligands binding to TLRs or IL-1 receptor agonists. These macrophages release both pro-inflammatory and anti-inflammatory cytokines, underscoring the complexity of the M2b response. In contrast, M2c macrophages are activated by glucocorticoids and IL-10 (88). Further research is required to elucidate the precise roles of each M2 macrophage subtype in disease pathogenesis.

An increase in M2 macrophage markers has been associated with remission in a mouse model of atherosclerosis, suggesting that an elevation in M2 macrophages may have therapeutic potential in atherosclerosis. This effect could prevent the progression of atherosclerosis through three distinct mechanisms. First, atherosclerotic lesions with increased M2 macrophage levels exhibit a reduced inflammatory response, leading to faster recovery from the disease (89). Furthermore, plaque cholesterol concentrations are reduced in this context. Second, M2 polarization induced by IL-13 enables macrophages to decrease collagen levels in lesions and inhibit VCAM-1-mediated monocyte recruitment, potentially improving atherosclerosis (88). Third, M2 macrophages can reduce the formation of necrotic cores in atherosclerotic lesions and phagocytose M1 macrophages, providing an additional mechanism to mitigate inflammation (90).

## 5 Natural products modulate lipid metabolism in relation to macrophages to ameliorate atherosclerosis

### 5.1 Natural products of plant origin

Numerous *in vitro* studies have demonstrated that plant-derived compounds regulate macrophage function and lipid metabolism, thereby improving atherosclerosis. Modulating cholesterol transport in macrophages is one of the mechanisms by which plant-derived compounds alleviate atherosclerosis. Ginkgetin exhibits a more targeted mode of action. It prevents ox-LDL from being absorbed by macrophages and from adhering to cellular components, thus inhibiting the formation of foam cells induced by ox-LDL. Additionally, it supports the use of natural chemicals for the development of therapeutic and/or chemopreventive molecules by inhibiting pro-atherogenic and inflammatory macrophage functions in a TRPV4-dependent manner (91). However, further investigation is necessary, as the current findings are limited to *in vitro* studies. There may be a significant correlation between macrophages and lipid metabolism through autophagy. Gynostemma saponin (GP), the primary bioactive component of Gynostemma pentaphyllum, a traditional Chinese medicine, also reduces the transformation of THP-1 cells into foam cells and macrophage uptake of ox-LDL (92). However, the role of Qi in these processes can be modulated by autophagy enhancers or inhibitors (93), suggesting that autophagy may be a critical pathway through which GP regulates macrophage lipid metabolism. Gynostemma saponin XVII (GP-17), a monomer of gynostemma glycoside isolated from Gynostemma, is associated with miR-182-5p and exhibits strong anti-atherosclerotic properties. In lipid-laden macrophages, GP-17 upregulates the expression of ABCA1, ABCG1, and miR-182-5p while downregulating HDAC9 expression. This promotes cholesterol efflux and inhibits lipid accumulation (94). Overexpression of HDAC9 or inhibition of miR-182-5p reversed the effects on ABCA1/G1 expression, lipid deposition, and pro-inflammatory responses (95). Ganoderic acid A (GAA) prevents ox-LDL-induced macrophage inflammation and lipid deposition in THP-1 cells by blocking Notch1/PPARγ/CD36 signaling, which may provide a theoretical framework for the clinical application of GAA in the treatment of atherosclerosis (96). Rosmarinic acid (RA) has proven benefits, including anti-inflammatory, antioxidant, antidiabetic, and cardioprotective effects. Key pathways for controlling macrophage cholesterol export include ABCA1 and ABCG1. Extensive research has shown that the differential regulation of JAK2/STAT3, JNK, and PKC-p38 underlies ABCA1 expression, while the regulation of JAK2/STAT3, JNK, and PKC-ERK1/2/p38 governs ABCG1 expression (97). Therefore, RA may control macrophage cholesterol export, potentially mitigating diabetes-related complications in atherosclerotic cardiovascular disease. The LXR/RXR signaling pathway is essential for macrophage lipid metabolism and the signaling of plant-derived compounds that can reduce atherosclerosis (98). Quercetin may reduce lipid accumulation in macrophages by enhancing cholesterol efflux, a process mediated by the LXR/RXR signaling pathway, as demonstrated by *in vitro* and computational studies (99). Similar *in vitro* and computational findings suggest that populin, a dietary flavonoid, can inhibit THP-1 monocyte migration and regulate cholesterol efflux from macrophages via the LXR/RXR pathway (100). This could reduce the risk of stroke or brain damage and prevent cellular infiltration in atherosclerotic plaques (101). Furthermore, PPARγ, a key protein involved in macrophage lipid metabolism, is an important target for plant-derived substances that mitigate atherosclerosis. In ApoE mice, the essential oil chemistry of Fructus Alpinia zerumbet (EOFAZ), known for its anti-atherogenic properties, directly binds to PPARγ, preventing its ubiquitination and enhancing its stability. This interaction activates the PPARγ-LXRα-ABCA1/G1 pathway, which inhibits foam cell formation from macrophages. By upregulating PPARγ/LXRα expression, polymethoxyflavonoids (PMFs) from grapefruit pericarp also promote cholesterol efflux from foam cells. In an alternative mechanism, this effect is achieved through the ABCG1/SRB1 pathway. Additionally, PMFs downregulate the expression of SRA1/CD36, thereby preventing lipid uptake and more effectively hindering lipid accumulation and foam cell formation in macrophages (102). This anti-atherosclerotic action of PMFs could offer new insights for their development in medical applications. Similarly, Guang Chen Pi regulates lipid metabolism via the PPARγ-LXRα-ABCG1/SRB1 pathway, inhibiting RAW264.7 foam cell production by blocking lipid uptake and stimulating HDL-mediated cholesterol efflux, showing anti-atherosclerotic potential (103). Baicalein, on the other hand, also enhances PPARγ expression, promotes cholesterol efflux, and prevents lipid accumulation. Important mechanisms in the regulation of macrophage lipid metabolism include ABCA1, ABCG1, and LXRα (104). The multi-target modulation of baicalein holds considerable promise for the treatment of atherosclerosis.

Animal experiments have further highlighted the potential role of plant-derived products in modulating atherosclerosis. Ethyl gallate (EG) was found to prevent early atherogenesis by influencing plaque lipid composition and macrophage infiltration. In terms of lipid regulation, treatment of macrophages with EG (20 μM) increased cellular cholesterol efflux to high-density lipoproteins (HDL) and reduced net lipid accumulation of ox-LDL (105, 106). Similarly, berberine has been shown to regulate ox-LDL uptake and cholesterol efflux, thereby inhibiting foam cell formation. This process may involve the upregulation of ATP-binding cassette transporter proteins via the activation of the Nrf2/HO-1 signaling pathway in human macrophages and the suppression of AP-1 activity to reduce scavenger receptor expression (107).

Mangiferin (MGF), a naturally occurring polyhydroxypolyphenol and C-glucosylflavonoid extracted from Mangifera indica L. (Anacardiaceae), has demonstrated significant hypoglycemic and hypolipidemic effects (95). Autophagy, which plays a role in regulating macrophage lipid metabolism, has been implicated in the atheroprotective actions of MGF and T0, which promote macrophage cholesterol efflux through AMPK-dependent autophagy. By focusing on autophagy, MGF may contribute to reducing atherosclerosis (95). Additionally, quercetin has been shown to reduce cholesterol absorption by preventing the phenotypic transition of vascular smooth muscle cells (VSMCs) into macrophage-like cells, thus limiting foam cell formation and delaying atherosclerosis progression (96). Chuan Chenopodium, also derived from citrus fruits, inhibits lipid absorption and foam cell formation in RAW264.7 cells. The PPARG signaling pathway is closely associated with this inhibitory effect. *in vivo* experiments have shown that NOB significantly reduces intra-arterial lipid buildup, macrophage infiltration, and CD36 expression (108). Furthermore, crocetin (CRO) has been reported to downregulate total cholesterol (TC) and cholesterol esters (CE), effectively limiting lipid droplet formation and activating PPARγ/LXR-α to facilitate reverse cholesterol transport, thereby ameliorating atherosclerosis (99).

To enhance its anti-atherosclerotic actions, EOFAZ can also modify the inflammatory microenvironment and lipid metabolism (101). PPARγ overexpression has been linked to the modulation of cholesterol transit and accumulation in atherosclerotic lesions through cruciferous vegetable-derived turnip thiols (SFN), an isothiocyanate. SFN regulates Nrf2, ABCA1/G1, and CD36, reducing lipid buildup, oxidative stress, and mitochondrial damage in macrophages derived from THP-1 cells (109). P. corylifolia L. (PFE) seed flavonoids similarly enhance ox-LDL-induced foam cell production by inducing cholesterol efflux via PPARγ-ABCA1/ABCG1. PFE also improved serum lipid profiles and reduces inflammatory markers in HFD-induced LDLR-/- mice, decreasing the size of atherosclerotic lesions and macrophage infiltration into the aortic root plaque (110).

Plant-derived chemicals offer multiple mechanisms for improving atherosclerosis. Salvia divinorum inhibits ox-LDL-induced lipid accumulation in foam cells. SIRT1 knockdown blocks the SIRT1/NRF2/GPX4 pathway, exerting a protective effect against lipid accumulation, suggesting that SIRT1 could be a viable target for atherosclerosis (111). Isodon rubescens (Hemsl.) winter grass methylin can inhibit foam cell formation by increasing lipid efflux proteins and decreasing lipid absorption proteins in macrophages. Furthermore, the stability of Nrf2 and suppression of NLRP3 are associated with the atheroprotective action of dong quai meconin in ApoE-/- mice, highlighting its potential as a therapeutic agent for atherosclerosis (112).

Natural bioactive substances, such as millet shell polyphenols (MSPs), reduce atherosclerosis by modulating macrophage lipid metabolism and inflammatory responses. MSPs inhibit macrophage STAT3 and NF-κB expression, thereby reducing lipid phagocytosis and the release of inflammatory cytokines such as TNF-α and IL-1β, preventing foam cell formation (113). Several plant-derived chemicals also influence miRNAs, which are implicated in various disorders. Curcumin, at an optimal dose, decreases foam cell production by THP-1 macrophages, reduces intracellular lipid levels, and enhances cholesterol efflux. It increases SIRT6 expression while suppressing miR-125a-5p, a miRNA that negatively targets SIRT6. The biological effects of curcumin are partially restored by overexpressing SIRT6, counteracting the inhibitory effects of miR-125a-5p mimics. Additionally, ginsenoside (Rb2), a bioactive component isolated from ginseng, binds to miR-216a and counteracts its effects under ox-LDL conditions, limiting lipid uptake and promoting cholesterol efflux. Rb2 also inhibits the Smad3/nuclear factor kappa B inhibitor alpha pathway, preventing miR-216a-mediated inflammatory responses. Thus, Rb2 may be a promising therapeutic agent for atherosclerosis, attenuating lipid accumulation, M1 macrophage polarization, and atherosclerotic plaque formation (114).

Gynostemma saponin XVII (GP-17), a monomer of gynostemma glycoside isolated from gynostemma, is associated with miR-182-5p and exhibits strong anti-atherosclerotic properties. In lipid-laden macrophages, GP-17 upregulates ABCA1, ABCG1, and miR-182-5p expression while downregulating HDAC9. This promotes cholesterol efflux and inhibits lipid accumulation. The reversal of ABCA1/G1 expression, lipid deposition, and pro-inflammatory responses by overexpressing HDAC9 or inhibiting miR-182-5p further emphasizes the potential of GP-17 as a therapeutic agent. However, further research is needed to explore the full impact of miR-182-5p on atherosclerosis and macrophage lipid metabolism (94).

### 5.1 Herbal compounding

PPAR-LXR is a prominent research topic in the use of herbal compounds to treat atherosclerosis by modulating macrophage responses and lipid metabolism. By stimulating the AMPK-PPARγ-LXRα-ABCA1 pathway, Tiao Gan Dao Zhuo Fang (TGDZF) reduces atherosclerosis and promotes cholesterol efflux from macrophages. Administration of TGDZF improved lipid profiles in ApoE mice and decreased both the extent of aortic plaque and lipid accumulation in aortic plaques and hepatocytes. Further research revealed that its effectiveness was associated with increased cholesterol efflux rate, as well as enhanced expression of PPARγ, LXRα, and ABCA1 mRNA and protein, alongside increased AMPKα1 phosphorylation (115). As demonstrated by a reduction in aortic atherosclerotic plaques in HHD-fed ApoE mice, lipid levels, and serum TMAO levels, QXXZF significantly mitigated the progression of atherosclerosis. The reduction in lipid levels, serum TMAO levels, and aortic atherosclerotic plaque in ApoE mice fed a high-fat diet was indicative of this. Moreover, QXXZF effectively inhibited foam cell formation in BMDM and TMAO-stimulated RAW264.7 macrophages, as well as in ox-LDL-induced macrophages. Additionally, QXXZF increased the expression of genes associated with cholesterol efflux, such as PPARγ, LXRα, ABCA1, and ABCG1, to facilitate reverse cholesterol transport (RCT) in macrophages. Mechanistic studies indicated that QXXZF affects cholesterol metabolism by blocking the nuclear factor kappa B (NF-κB) axis mediated by TLR4. Notably, the effects of QXXZF on macrophages were reversed by TLR4 knockdown (116).

In a similar context, using the Shenhong Tongluo (SHTL) formula as a pretreatment prevented the formation of reactive oxygen species (ROS) and reversed the increase in inflammatory factors such as TNF-α and IL-6. Moreover, SHTL pretreatment reduced ox-LDL-induced lipid accumulation in macrophages. By activating the PPAR-γ/LXR-α/ABCA1 pathway, SHTL pretreatment reduced macrophage inflammation and lipid accumulation (117). These findings may provide insights into the mechanisms by which SHTL prevents the progression of atherosclerosis. The extract from Shenlian (SL), composed of extracts from Salvia miltiorrhiza Bunge and Andrographis paniculata (Burm.f.) Nees—two herbs commonly used in traditional Chinese medicine (TCM) to treat atherosclerosis by removing blood stasis and dispelling heat—significantly reduced endoplasmic reticulum (ER) stress in a macrophage model of lipid excess. This reduction ultimately prevented ox-LDL-induced death in foam cells. The protective effect of SL extracts on macrophages was significantly diminished when the ER stress inhibitor 4-phenylbutyric acid (4-PBA) blocked ER stress. Further investigation revealed that the beneficial effects of SL extracts on macrophages require the functionalization of the LAL-LXRα axis, as demonstrated by the use of selective antagonists against LAL and LXRα (118).

Beyond the PPAR-LXR pathway, plant extracts also reduce the risk of atherosclerosis by regulating macrophages and lipid metabolism through several other mechanisms. Huanglian Jiedu Tang (HLJDD), by increasing the expression of SLC2A1, can control macrophage cytotoxicity and enhance the stability of atherosclerotic plaques. Simultaneously, the administration of HLJDD resulted in a significant reduction in TG, TC, and LDL-C levels, as well as an increase in HDL-C levels in mice, demonstrating its effectiveness. However, the relationship between alterations in lipid levels and macrophage cytotoxicity remains unclear (119). One of the key stages in the development of atherosclerosis is the necrosis of the plaque cores, caused by macrophage phagocytosis of lipid particles that undergo oxidative modification, leading to foam cell formation and mass cell death (120). ZTLT has been shown to limit foam cell formation and reduce lipid levels by preventing vascular inflammation and macrophage pyroptosis through the Piezo1/NLRP3 signaling pathway, thus slowing the progression of atherosclerosis (121).

ATM can regulate postprandial cholesterol levels by controlling lipid uptake, hydrolysis, and cholesterol export in macrophages. Animal studies show that SWTX significantly reduces atherosclerotic plaque formation, raises postprandial HDL cholesterol levels, increases the percentage of ATMs expressing Tim4 and CD36, and boosts ATM lipid absorption and lysosomal activity. The effects of SWTX are dependent on increased lysosomal activity, as shown by the lysosomal function inhibitor chloroquine limiting the impact of SWTX. Additionally, certain bioactive compounds in SWTX can enhance lysosomal activity *in vitro* (122). Similarly, the autophagy agonist Torin1, like DLT, reduced intracellular lipid accumulation in BMDM. Furthermore, DLT decreased p62 accumulation in RAW264.7 cells and increased the expression of the autophagy-related protein LC3II. DLT also suppressed the phosphorylation of p-Akt, p-PI3 K, and p-mTOR, leading to upregulation of autophagy in RAW264.7 cells. Overall, these findings suggest that DLT promotes macrophage autophagy by inhibiting the PI3 K/Akt/mTOR signaling pathway, which may help reduce foam cell formation and improve atherosclerosis (123).

### 5.2 Other natural products

In addition to plants, natural products derived from microbial and animal sources also hold potential for ameliorating atherosclerosis. Equisetin (EQST), a semi-estrogenic molecule, has been shown to possess antibacterial, anti-inflammatory, lipid-lowering, and weight-loss activities. It was isolated from a fungus related to sea sponges (124–126). By inhibiting lipid absorption, foam cell formation, and macrophage inflammatory responses, EQST demonstrates anti-atherosclerotic properties *in vitro* and ultimately reduces the impact of a HFD on atherosclerosis *in vivo* (127). Neofusicoccum parvum JS-0968, an endophytic fungus isolated from Vitex rotundifolia, was found to produce (3R)-5-hydroxymellein, a secondary metabolite, through chemical analyses of its cultures. This compound reduces LDL and HDL oxidation by blocking lipid peroxidation, lowering negative charge, reducing hyperchromism and carbonyl content, and limiting the aggregation of apolipoprotein ApoA-I (A-I) and the fragmentation of apoB-100. Furthermore, (3R)-5-hydroxymellein significantly decreases foam cell formation induced by ox-LDL. Overall, these findings suggest that (3R)-5-hydroxymellein may serve as a viable preventive measure against atherosclerosis by substantially inhibiting foam cell production and the oxidation of LDL and HDL (128).

Antimicrobial peptides (AMPs) derived from frog skin have been shown to enhance the survival of foamy macrophages generated by ox-LDL and reduce the production of intracellular lipid droplets, total cholesterol, and cholesteryl esters (CE). By decreasing the protein expression of CD36, which regulates ox-LDL uptake, while having no effect on the expression of the exocytosis proteins ATP-binding cassette subfamily A/G member 1 (ABCA1/ABCG1), frog skin AMPs prevent foam cell formation (129). The RJ proteins also demonstrated a significant, concentration-dependent inhibition of macrophage proliferation in experiments conducted on cultured mouse J774A.1 macrophages, irrespective of the presence of LDL or ox-LDL. However, these proteins had no impact on lipid accumulation within macrophages. These findings suggest that RJ proteins, including degradation products such as MRJP1/MRJP3 and royalisin, a potent antimicrobial protein found in royal jelly (RJ), may offer therapeutic advantages for atherosclerosis by reducing plaque inflammation. Further research on these RJ proteins may lead to the discovery of novel anti-atherosclerotic therapies (130).

The application of cutting-edge technologies has revitalized the potential of natural products. Prussian blue nanoparticles (PB NPs) encapsulated in bionic membranes have been employed in atherosclerosis treatment by leveraging the actions of procyanidin (PC) and artemisinin (ART) on lipid inflow and outflow in macrophages, two key processes contributing to plaque formation. The generated nano-complexes exhibited strong scavenging effects on ROS and NO *in vitro*, which were followed by suppression of the NF-κB/NLRP3 pathway and a reduction in lipid inflow. Meanwhile, by significantly enhancing the AMPK/mTOR/autophagy pathway, these complexes considerably decreased ox-LDL absorption and internalization while promoting cholesterol efflux (131). Curcumin, by polarizing macrophages, improves the stability of atherosclerotic plaques. However, its nonspecific targeting makes its clinical application challenging. The attachment of 125I-ION to lipids bearing phagocytic “eat me” signals triggers macrophages to phagocytose multifunctional lipid nanoparticles (MLNP), which selectively accumulate on unstable plaques and can be precisely detected and visualized by SPECT and MRI. Moreover, MLNP demonstrates a high efficacy in delivering curcumin and 125I-ION to macrophages, which, in turn, leads to significant polarization of macrophages from M1 to M2 phenotypes (132).

## 6 Result and discussion

Different types of lipoproteins have varying effects on lipid metabolism, and disruptions in lipid metabolism are a major factor in the development of atherosclerosis. The primary risk to vascular health is LDL, which triggers complex metabolic processes that contribute to the formation of atherosclerotic plaques. Therefore, a key strategy for mitigating atherosclerosis is to regulate the expression levels and function of lipoproteins. Macrophage-lipid metabolism interactions are intricately involved in all aspects of lipid metabolism, including fatty acid β-oxidation, cholesterol metabolism, lipogenesis, catabolism, uptake, and transport (28). Macrophages and lipid metabolism interact through a range of enzymes and signaling pathways, influencing the development of atherosclerosis. Macrophages play a crucial role in the formation and detachment of atherosclerotic plaques, with their recruitment and proliferation contributing to these processes, which negatively impact patients’ quality of life and health (71–73). Atherosclerosis is influenced by distinct macrophage types. M1 macrophages, through their pro-inflammatory response, indicate vascular damage, accelerating the progression of atherosclerosis (85). In contrast, M2 macrophages exhibit anti-inflammatory properties that help reduce atherosclerotic plaques (89). Natural products have shown potential in treating atherosclerosis by modulating lipid metabolism and macrophage function through similar signaling pathways, including various miRNAs, LXR/RXR signaling, PPAR/LXR signaling, and cholesterol uptake and efflux. These natural products hold significant potential for reducing atherosclerosis, with their main mechanisms of action involving the modulation of macrophages and lipid metabolism.

Despite ongoing research clarifying the relationship between lipid metabolism and macrophages, several questions remain unanswered. Most current studies focus on specific molecular pathways, such as PPAR/LXR signaling, LXR/RXR signaling, cholesterol absorption and efflux, and certain lipid metabolism-related genes. While these molecular processes primarily mediate the link between lipid metabolism and macrophages, further research is needed to fully understand these interactions. Macrophage classification has expanded beyond the traditional M1 and M2 subtypes and continues to evolve. The pathophysiological impact of macrophages on atherosclerosis, along with their interactions with lipid metabolism in other subtypes, remains poorly understood. Although the relationship between natural compounds and modulation of lipid metabolism and macrophages is actively being studied, most findings are still limited to animal models, with some results validated only *in vitro* or through computational models. The safety of these natural compounds requires further investigation, and their clinical efficacy has yet to be established.

Atherosclerosis treatment continues to be a global challenge. Future research must offer novel insights and develop a fresh theoretical framework for treating atherosclerosis. First, more gene-level research is needed to deepen our understanding of macrophages’ role in lipid metabolism. Second, to expand the study of macrophages and lipid metabolism and better define their function in atherosclerosis, researchers should explore additional macrophage subtypes beyond M1 and M2. In parallel, the research on natural products should be accelerated to ensure their clinical applicability, with thorough evaluations of their safety. Lastly, advancements in technology have enhanced the efficacy and precision of natural products, and future studies should combine new technologies with natural products to revitalize their potential applications.
